# Laparoscopic sigmoidectomy in a case of sigmoid colon cancer with situs inversus totalis

**DOI:** 10.1111/ases.12483

**Published:** 2018-03-30

**Authors:** Takashi Takeda, Naotsugu Haraguchi, Ayumi Yamaguchi, Mamoru Uemura, Masakazu Miyake, Michihiko Miyazaki, Masataka Ikeda, Mitsugu Sekimoto

**Affiliations:** ^1^ Department of Surgery National Hospital Organization Osaka National Hospital Osaka Japan; ^2^ Department of Gastroenterological Surgery Graduate School of Medicine, Osaka University Osaka Japan

**Keywords:** Laparoscopic sigmoidectomy, sigmoid colon cancer, situs inversus totalis

## Abstract

Situs inversus totalis (SIT) is a rare anatomic anomaly in which organs in the chest and abdomen exist in a mirror image reversal of their normal positions. SIT can complicate surgical procedures, and few reports have described laparoscopic surgery for colorectal cancer in patients with SIT. Here, we report a case of successful laparoscopic surgery in a patient with SIT and sigmoid colon cancer. Laparoscopic sigmoidectomy involved colonic mobilization with high ligation of the inferior mesenteric vessels and complete mesocolic excision. The operating surgeon stood on the patient's left side, opposite the normal location for sigmoidectomy. By placing a 12‐mm trocar in the left iliac fossa and using an automatic endoscopic linear stapler, the operating surgeon was able to perform left‐handed colon resection without having to change position or move the laparoscopic monitor mid‐procedure. An automatic endoscopic linear stapler is useful for laparoscopic left‐side colon surgery in a patient with SIT.

## Introduction

Situs inversus totalis (SIT) is a rare congenital anomaly in which the organs in the chest and abdomen are located in a mirror image reversal of their normal positions. SIT occurs in 1 of every 10 000–50 000 people 1, and co‐occurrence of SIT and colon cancer is even rarer. Laparoscopic colorectal surgery for SIT requires procedural modifications because of the mirror image organ positioning. However, few such cases have been reported. The English‐language literature includes only one previous description of laparoscopic sigmoidectomy for sigmoid colon cancer in a patient with SIT 2, and this report indicated the effectiveness of changing the positions of the operating surgeon and assistant.

Here, we report a case of successful laparoscopic sigmoidectomy in a patient with SIT and sigmoid colon cancer. Using an automatic endoscopic linear stapler and altering the monitor and port arrangement enabled performance of the operation without mid‐procedure changes in the positions of the operating surgeon and assistant.

## Case Presentation

**Figure 1:**
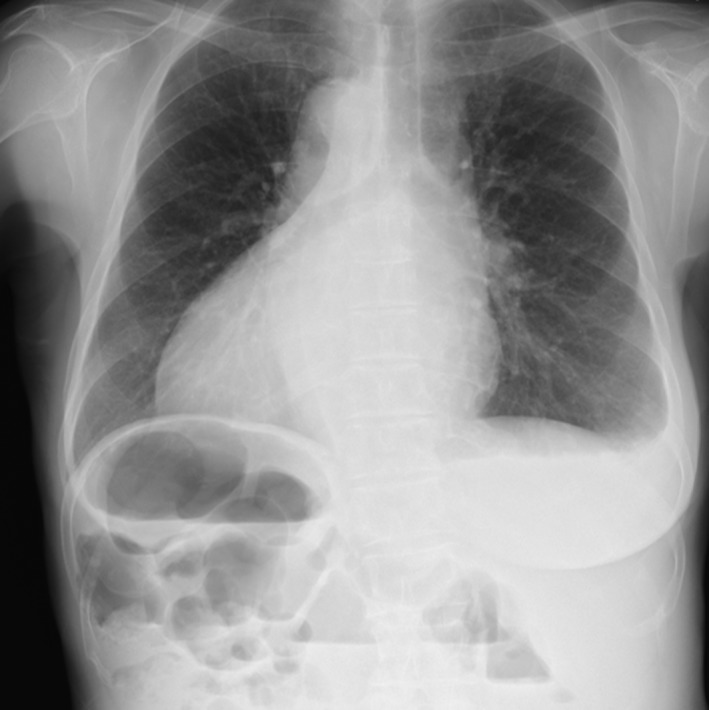
Chest radiography showing dextrocardia.

**Figure 2:**
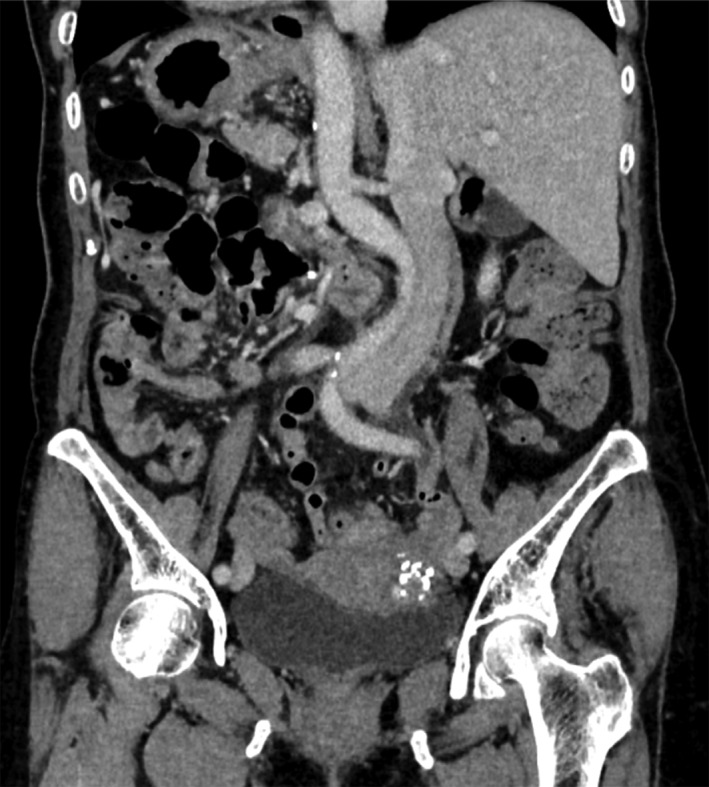
CT image showing complete transposition of the thoracic and abdominal viscera.

A 72‐year‐old woman was admitted because of fecal occult blood and severe anemia. Colonoscopy revealed an advanced obstructive tumor in the sigmoid colon, which was diagnosed as a well‐differentiated adenocarcinoma by biopsy. The patient had had no previous abdominal surgeries. Laboratory examination showed severe iron‐deficiency anemia (hemoglobin, 4.7 g/dL), elevated serum carcinoembryonic antigen (16.0 ng/mL; reference range, 0–4 ng/mL), and normal carbohydrate antigen (CA19‐9; 9 U/mL; reference range, 0–37 U/mL). Chest radiograph revealed dextrocardia and a right subphrenic gastric bubble (Figure 1). CT confirmed SIT, showing complete transposition of the thoracic and abdominal viscera (Figure 2). CT also revealed a tumor in the sigmoid colon, with no lymph node metastasis or distant metastasis (cT3, cN0, cM0 cStage IIA according to the UICC‐TNM classification) 3. We determined that curative operation with laparoscopic sigmoidectomy was feasible.

**Figure 3:**
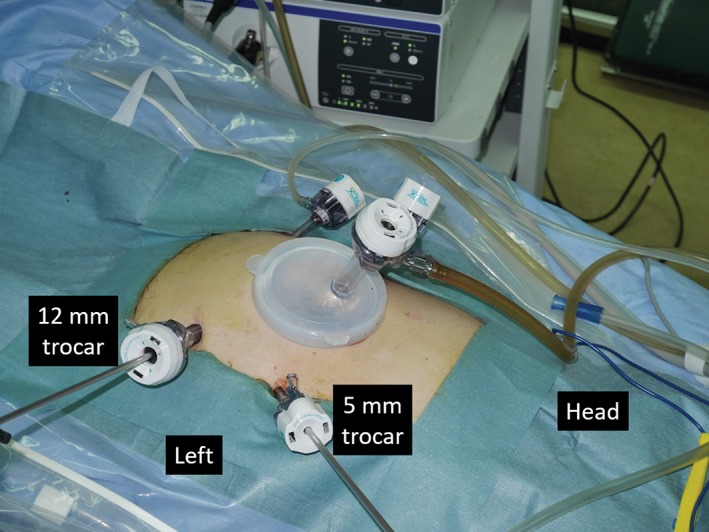
A 12‐mm trocar was placed in the left iliac fossa.

For surgery, the patient was placed in the lithotomy position under general anesthesia. In a reversal of the normal setup, the operating surgeon and scopist were situated on the patient's left side, and the first assistant on the right. First, a lap protector mini (Hakkou Shoji, Nagano, Japan) was inserted through a 30‐mm transumbilical incision. Next, an EZ access port (Hakkou Shoji) was mounted onto the lap protector mini, and a 12‐mm camera port was placed through the EZ access port. For the operating surgeon, a 12‐mm trocar was placed in the left iliac fossa and a 5‐mm trocar in the left flank. For the first assistant, 5‐mm trocars were placed in the right iliac fossa and the right flank (Figure 3). Abdominal air pressure was set to 10 mmHg.

**Figure 4:**
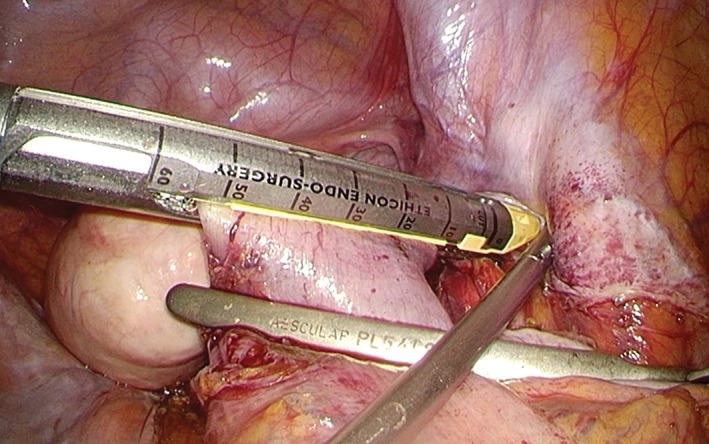
Intraoperative view showing colon division with an automatic endoscopic linear stapler held in the surgeon’s left hand.

Via a medial approach, the sigmoid colon was mobilized with an ultrasonic harmonic scalpel. The right ureter was confirmed on the dorsal side. The inferior mesenteric artery was identified and isolated. The inferior mesenteric artery was clipped with endoscopic vascular clips and then divided (D3 lymph node dissection). Next, the inferior mesenteric vein was divided. After mesenteric treatment was performed on the anal side 10‐cm from the tumor, detachable forceps were applied to the immediate side of the line to be dissected, and the intestinal tract was washed from the anus. The distal side was divided using an endoscopic linear stapler (Powered ECHELON FLEX® ENDOPATH Stapler; Ethicon, Tokyo, Japan), which was inserted from the 12‐mm trocar in the left iliac fossa (Figure 4). The pneumoperitoneum was stopped, and then the colon was divided at the proximal side of the tumor. After the pneumoperitoneum was restored, anastomosis was completed using a circular stapler (CDH29; Ethicon).

The operation time was 195 min with low blood loss. The postoperative course was uneventful, with no complications. Pathological examination revealed a well‐differentiated adenocarcinoma: 55 × 45 mm, pT3 (SS), int, INFb, ly2, v0, PN0, pN0 (0/35), pPM0 (125 mm), pDM0 (105 mm), pRM0, pStage IIA.

## Discussion

SIT is a rare anatomic anomaly in which the left and right aspects of the thoracic and intra‐abdominal organs are inverted. Patients with SIT may have a higher risk of cancer 4, and laparoscopic procedures are considered more difficult because of the mirror‐image anatomy. When surgeons operate on patients with SIT, it is important that they pay attention to anatomic variations. CT angiography is useful for clarifying the anatomy. Prior reports described the effectiveness of the surgeon switching from the patient’s left to right side during a pelvic procedure 2, 5. However, when the surgical operator and assistant change position mid‐procedure, this requires the monitor and devices to be re‐set, slowing down the operation. Another report proposed that left‐handed surgical operators have advantages during laparoscopic procedures in patients with SIT 6.

In the present case, the operating surgeon, scopist, first assistant, and trocars were positioned opposite of the conventional positions. When the surgical operator stands to the patient’s left, the port in the left upper abdomen or left iliac fossa becomes the approach port for colon resection. For a right‐handed operator, a port in the left upper abdomen may be easiest for colon resection because colorectal dissection requires firmly grasping the dissection device but delicate and gentle device use. However, using the left upper abdominal port for colorectal dissection requires retracting the colon toward the head with the forceps in the left hand. This situation requires the intersection of the forceps and the linear stapler, making the colorectal dissection procedure difficult. In contrast, using the port in the left iliac fossa for colorectal dissection does not require intersection between the forceps and the linear stapler, allowing for the linear insertion of the linear stapler perpendicularly to the intestinal axis.

An automatic endoscopic linear stapler (Powered ECHELON FLEX® ENDOPATH Stapler) was used in the present case. This made it easy for the surgeon to perform the operation left‐handed, without changing positions during surgery. The operative time and blood loss were comparable to those in orthotopic patients. Apart from the left‐handed use of an automatic endoscopic linear stapler, the procedure did not differ from usual surgery.

Here we described the performance of laparoscopic sigmoidectomy with radical lymphadenectomy in a patient with SIT and sigmoid colon cancer. We believe that laparoscopic sigmoidectomy is feasible in patients with SIT if adequate attention is paid to the anatomic anomaly. An automatic endoscopic linear stapler was useful for allowing a right‐handed surgeon to perform left‐handed colon division without changing position during the operation.

## Acknowledgments and disclosure

The authors thank Dr. Yoshinori Kodama, Dr. Kiyoshi Mori, and Dr. Masayuki Mano for the pathological diagnosis. The authors have no conflicts of interest to declare.

No ethical approval was required for this case report.

Written informed consent was obtained from the patient for publication of this case report and any accompanying images. Patient anonymity was maintained.
